# Preparation and High-Temperature Fracture-Sealing Performance of an Oil-Absorbing Swelling Gel for Lost-Circulation Control in Oil-Based Drilling Fluids

**DOI:** 10.3390/gels12090840

**Published:** 2026-09-14

**Authors:** Kun Zhang, Qiwei Liang, Xin Chen, Jing Yan, Dong Guo, Yongqiang Pan, Xiuyu Zhu, Xuntao Jiang, Jingbin Yang

**Affiliations:** 1 Engineering Technology Research Institute, Daqing Drilling Engineering Co., Ltd., Daqing 163413, China; 2 School of Petroleum Engineering, China University of Petroleum (East China), Qingdao 266580, China

**Keywords:** oil-based drilling fluid, oil-absorbing swelling gel, lost-circulation material, fracture sealing, viscoelasticity, artificial fracture

## Abstract

Lost circulation in fractured formations remains challenging for oil-based drilling fluids because conventional rigid bridging materials can be sensitive to fracture geometry and form porous seals. In this study, an oil-absorbing swelling gel plugging agent (OSGP) was prepared by water-in-oil emulsion free-radical polymerization. Its oil-induced swelling, strain-dependent viscoelasticity, and bidirectional fracture-sealing behavior were evaluated together with morphology and thermal mass-loss behavior. After 12 h in white oil at 120 °C, the particle volume increased by 19.2%. At 25 °C, OSGP showed an elastic-dominant response with strain-induced softening. In independent fracture-sealing tests at 120 °C, OSGP formed pressure-bearing seals in both 3 mm parallel and 3 mm-to-1 mm wedge-shaped steel fractures, with a maximum forward breakthrough pressure of 8.06 MPa. Comparison of forward and reverse tests showed that fracture geometry and flow direction strongly affected pressure-bearing behavior. The combined results support a proposed sealing process involving particle bridging, oil-induced swelling, deformation, and compaction. OSGP therefore shows potential for further evaluation as a lost-circulation material in fully formulated oil-based drilling fluids and rock fractures.

## 1. Introduction

The continued development of deep, ultra-deep, and unconventional oil and gas resources has increased the frequency of high-temperature and high-pressure conditions, fracture development, and wellbore instability during drilling. Oil-based drilling fluids are widely used in shale oil and gas wells, long horizontal sections, and complex formations because of their thermal resistance, lubricity, inhibition, and reservoir-protection performance [1,2,3]. Nevertheless, oil-based drilling fluids remain vulnerable to lost circulation in natural fractures, induced fractures, and weakly consolidated formations. Because these fluids are costly, losses not only consume large volumes of drilling fluid but may also induce wellbore pressure imbalance, borehole collapse, stuck pipe, and formation damage [2,3,4,5,6]. Developing a lost-circulation material that is compatible with oil-based drilling fluids, accommodates variations in fracture aperture, and forms a stable seal is therefore important.

Lost-circulation materials for fractured formations include rigid particles, fibers, flakes, composite bridging materials, polymer gels, curing resins, and expandable materials [7,8,9]. Belayneh and Aadnøy noted that conventional bridging materials rely mainly on particle-size distributions to form mechanical bridges in fractures. Although they are widely available and convenient to use, their sealing effectiveness depends strongly on fracture aperture, particle size, and particle morphology [10,11,12,13]. When fracture dimensions vary substantially or fluid erosion is strong, bridging seals may exhibit high porosity, inadequate density, and poor pressure-bearing stability [14,15,16,17]. Qiu et al. showed that polymer gels and resin-based materials can form continuous or semi-continuous structures within loss channels and therefore provide spatial adaptability and integral sealing [18]. However, their gelation time, curing window, and pumping safety can be affected by temperature, shear, and downhole-fluid composition [19,20,21,22,23]. Because oil-based drilling fluids have a continuous oil phase, materials that swell directly in oil are better matched to this fluid environment [24,25].

Oil-absorbing swelling gels provide a potential route for controlling losses of oil-based drilling fluids. The oleophilic polymer lost-circulation material prepared by Zhong et al. showed oil-absorption swelling and improved the sealing performance of an oil-based drilling fluid [24]. Studies of oil-absorbing gels and resins by Bai et al. further showed that these materials can participate in fracture sealing through oil absorption, swelling, deformation, and filling [25,26,27]. Structurally, such materials generally contain oleophilic segments, functional monomers, and crosslinkers. Oil molecules diffuse into the polymer network and expand its volume, while functional-monomer selection and crosslink-network design influence oil absorption, oil retention, and thermal stability [26,27].

A persistent challenge is to balance oil-absorption swelling with mechanical strength. A low crosslink density facilitates oil diffusion and segmental motion, thereby increasing swelling and flexible deformation, but it may also promote excessive deformation or washout under a pressure differential. A high crosslink density strengthens the network but can restrict segmental motion and oil diffusion, thereby reducing swelling [28]. Rubber nanogels and resin–gel–rubber blends provide related strategies for improving deformability and oil-phase adaptability [29,30]. Blended particulate LCM systems have also been investigated with respect to fracture sealing and seal integrity [31], while oil-absorbing rubber-based materials provide another related strategy [32]. Recent studies have developed polymer microspheres and submicron particles to improve sealing in microfractures and pores [33,34,35]. Other studies have incorporated inorganic nanoparticles or rigid fillers to improve the thermal and mechanical performance of oil-phase plugging materials [36,37]. Recent reviews have summarized advances in polymer gels for drilling and production engineering [38], while high-temperature polymer gels have also been developed for lost-circulation control [39]. Crosslinked polymer systems have further been reviewed in the context of lost-circulation control and wellbore strengthening [40]. However, fewer studies have examined how oil-induced swelling and deformation of particulate gels are related to sealing behavior in fractures with different geometries and flow directions.

Unlike approaches that rely mainly on oil absorption or rigid particle bridging, the present OSGP combines moderate delayed swelling, viscoelastic deformation, and particle bridging to adapt to fractures with different geometries. In this study, an oil-absorbing swelling gel plugging agent (OSGP) was prepared as a candidate lost-circulation material for oil-based drilling fluids. Single-factor screening was used to select the monomer composition, crosslinker dosage, surfactant system, bentonite dosage, polymerization temperature, and stirring speed. SEM, FTIR, TGA, swelling tests, and dynamic rheology were used to characterize the morphology, functional groups, thermal mass-loss behavior, oil-induced swelling, and viscoelastic response of OSGP. Pressure-bearing behavior was evaluated at 120 °C in a 3 mm constant-aperture fracture and a 3 × 1 mm wedge-shaped steel fracture using white oil as the carrier fluid. The main contribution of this study is the combined evaluation of oil-induced swelling, viscoelastic behavior, and bidirectional fracture-sealing performance of millimeter-scale OSGP particles. By comparing constant-aperture and wedge-shaped fractures, the study further examines the effects of fracture geometry and flow direction on seal formation and stability. Based on these results, a bridging–swelling–compaction sealing model was proposed.

## 2. Results and Discussion

### 2.1. Preparation and Formulation Screening of OSGP

**Figure 1:**
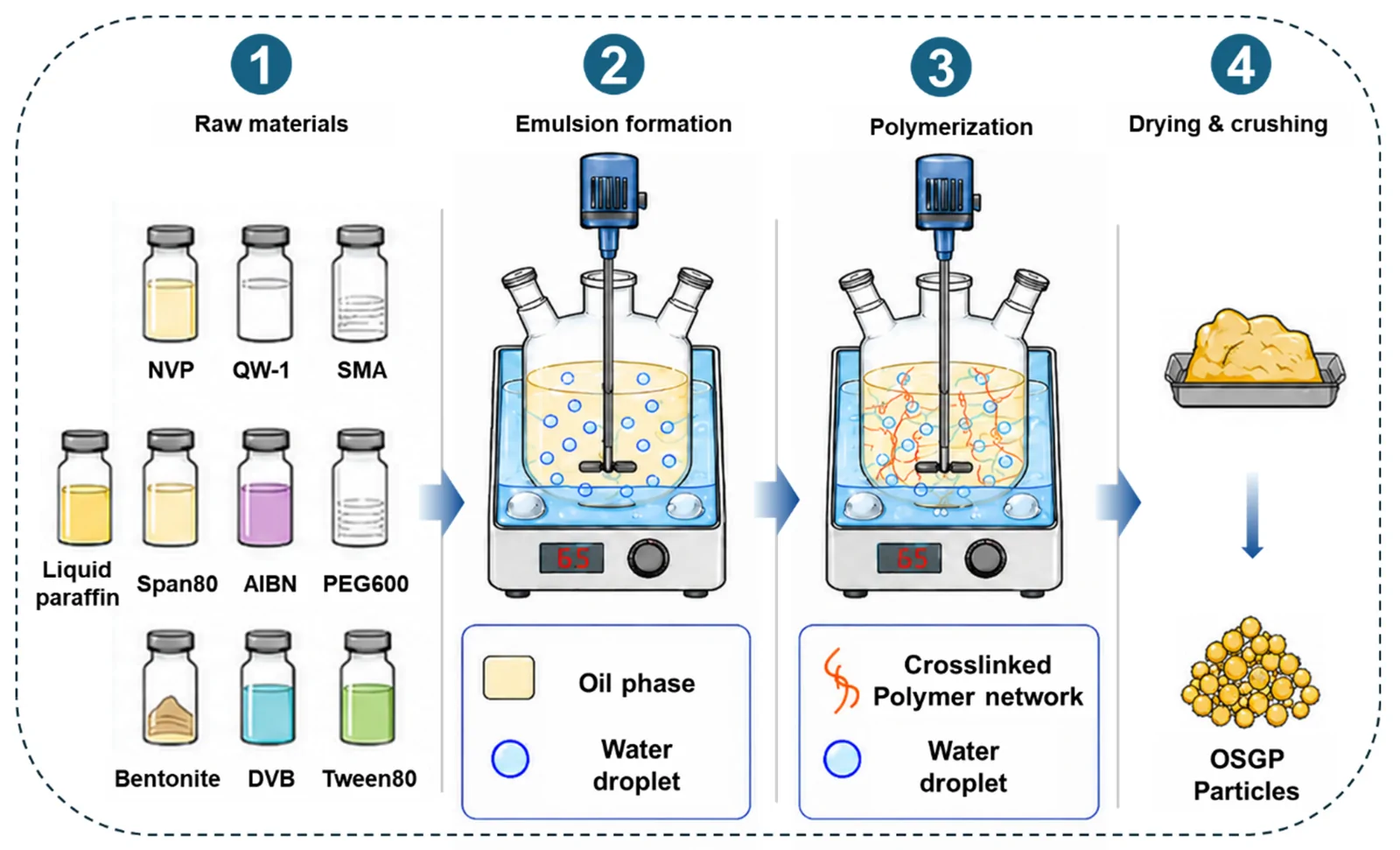
Schematic illustration of the preparation process of the oil-absorbing swelling gel plugging agent (OSGP).

OSGP was prepared by W/O emulsion free-radical polymerization to obtain oil-absorbing swelling gel particles for sealing fractures during drilling with oil-based fluids. The process comprised W/O emulsion formation, free-radical polymerization and crosslinking, gel curing, drying, crushing, and sieving (Figure 1). This route produced a crosslinked gel network in an oil-continuous system, allowing limited oil-absorption swelling while maintaining particulate integrity.

N-vinylpyrrolidone (NVP) and an acrylate monomer designated QW-1 served as the principal functional monomers. QW-1 is a commercial functional acrylate monomer containing polymerizable acrylate groups. It was used as a co-monomer to adjust the flexibility and swelling behavior of the polymer network. A styrene–maleic anhydride copolymer (SMA) was used to adjust oil affinity and network structure; divinylbenzene (DVB) served as the crosslinker; azobisisobutyronitrile (AIBN) initiated free-radical polymerization; Span 80 and Tween 80 stabilized the W/O emulsion; polyethylene glycol 600 (PEG600) acted as a pore-forming/structure-regulating component; and bentonite provided inorganic reinforcement. The selected formulation and processing conditions are listed in Table 1.

### 2.2. Effects of Preparation Parameters in Single-Factor Screening

**Figure 2:**
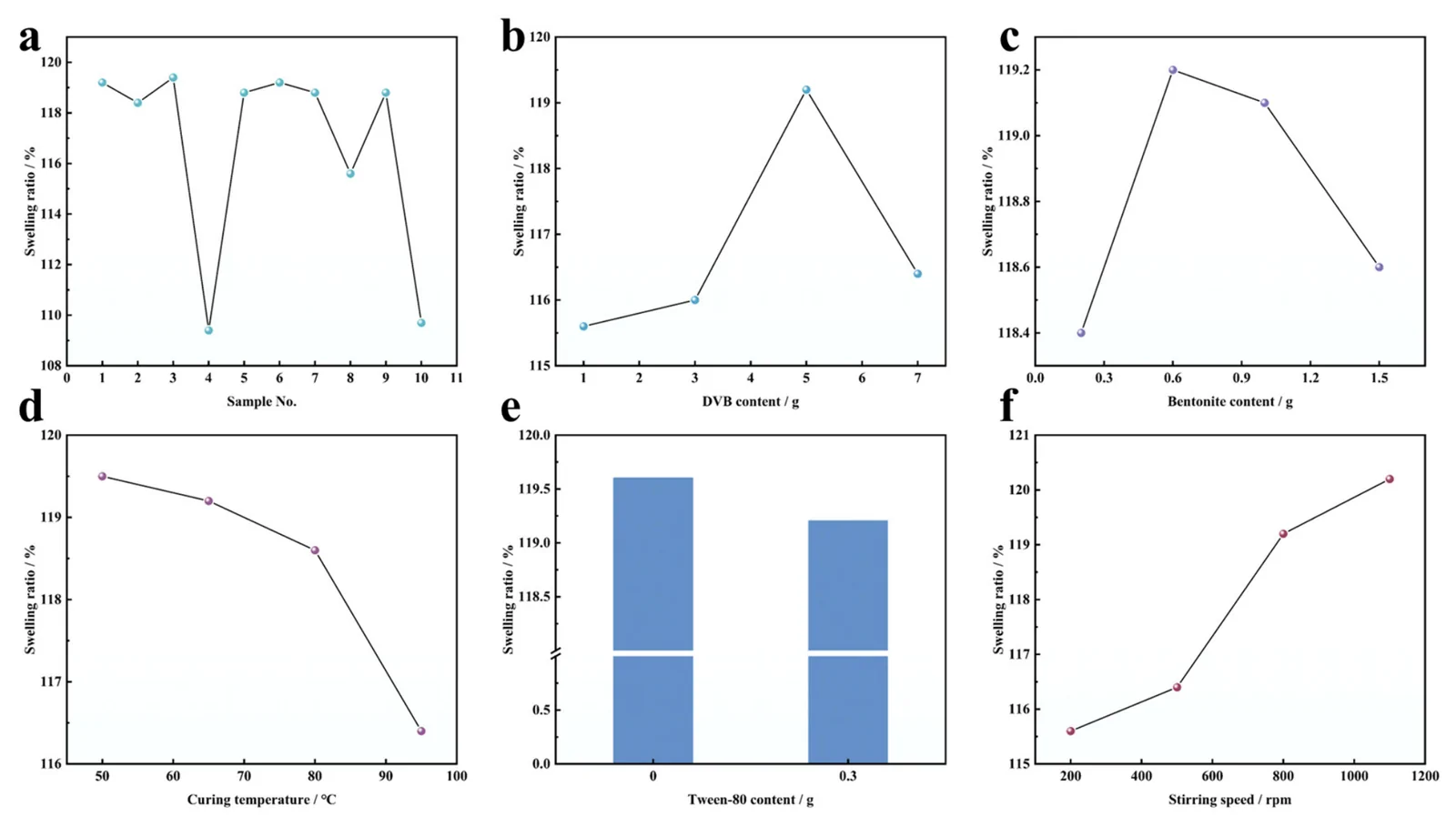
Effects of preparation parameters on the swelling ratio of OSGP after 12 h in white oil at 120 °C: (**a**) monomer composition; (**b**) DVB content; (**c**) bentonite content; (**d**) polymerization temperature; (**e**) Tween 80 content; and (**f**) stirring speed.

Monomer composition, DVB dosage, Tween 80 dosage, bentonite dosage, polymerization temperature, and stirring speed were varied individually, and the swelling ratio was used as the primary screening indicator (Figure 2 and Table 2). The viscoelastic behavior of the selected formulation is discussed separately in Section 2.5.

Overall, the preparation parameters affected swelling and network stiffness in different ways. The final formulation was selected on the basis of the swelling responses observed under the tested single-factor conditions and was subsequently evaluated for viscoelastic behavior and fracture-sealing performance.

Monomer composition directly affected network structure and swelling in oil. The tested NVP/QW-1/SMA formulations formed gels with different swelling responses in white oil at 120 °C. For most formulations, the swollen volume was approximately 1.18–1.19 times the initial volume, whereas compositions outside the selected range showed less swelling. Based on the swelling response observed in the single-factor screening, an NVP/QW-1/SMA composition of 20 g/10 g/12 g was selected for subsequent characterization.

The swelling ratio increased as the DVB dosage increased to 5 g and decreased at higher dosages. This nonmonotonic trend reflects the balance between network integrity and segmental mobility. Therefore, 5 g of DVB was selected.

With 3 g of Span 80, adding 0.3 g of Tween 80 caused only a small change in the swelling ratio. Tween 80 was retained as a co-emulsifier in the selected formulation.

Bentonite was included as an inorganic reinforcing component. The swelling ratio varied only slightly over the tested dosage range, and 0.6 g produced the highest swelling ratio among the tested bentonite dosages. Therefore, 0.6 g was selected.

The swelling ratio generally decreased as the polymerization temperature increased. Although lower temperatures produced slightly higher swelling ratios, 65 °C retained a relatively high swelling ratio while providing a practical polymerization condition and was therefore selected.

The swelling ratio increased with stirring speed, but the increase from 800 to 1100 rpm was relatively small. Therefore, 800 rpm was selected to obtain a high swelling ratio without using the highest agitation intensity.

### 2.3. Morphological and FTIR Characterization of OSGP

Oil-absorption swelling and fracture sealing are related to the morphology and chemical structure of OSGP. Surface morphology is related to the interaction between OSGP particles and the oil phase, while FTIR was used to identify the principal functional groups. SEM was used to compare surface morphology before and after swelling, and FTIR spectroscopy was used to identify the principal functional groups.

#### 2.3.1. Surface Morphology Before and After Oil Exposure

**Figure 3:**
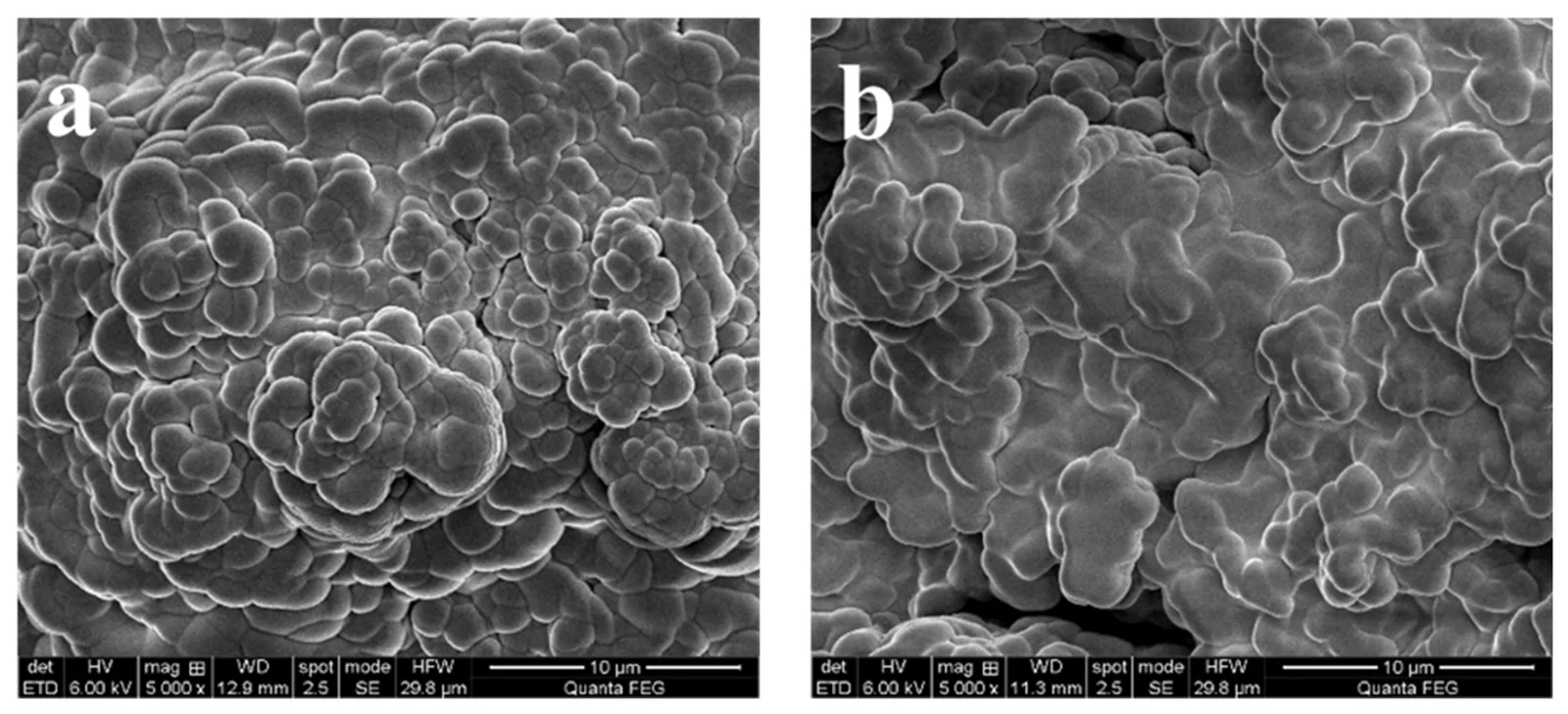
SEM images of OSGP before and after oil exposure: (**a**) dry OSGP before swelling; and (**b**) OSGP after swelling in white oil at 120 °C, followed by surface-oil removal and vacuum drying.

Before oil exposure, OSGP exhibited a rough and microscopically undulating surface (Figure 3a). After oil exposure and subsequent drying, the surface appeared smoother, while the particles remained intact without obvious collapse or fragmentation (Figure 3b). These observations indicate that oil exposure altered the external surface morphology of OSGP while the overall particle integrity was retained.

#### 2.3.2. FTIR Characterization of OSGP

**Figure 4:**
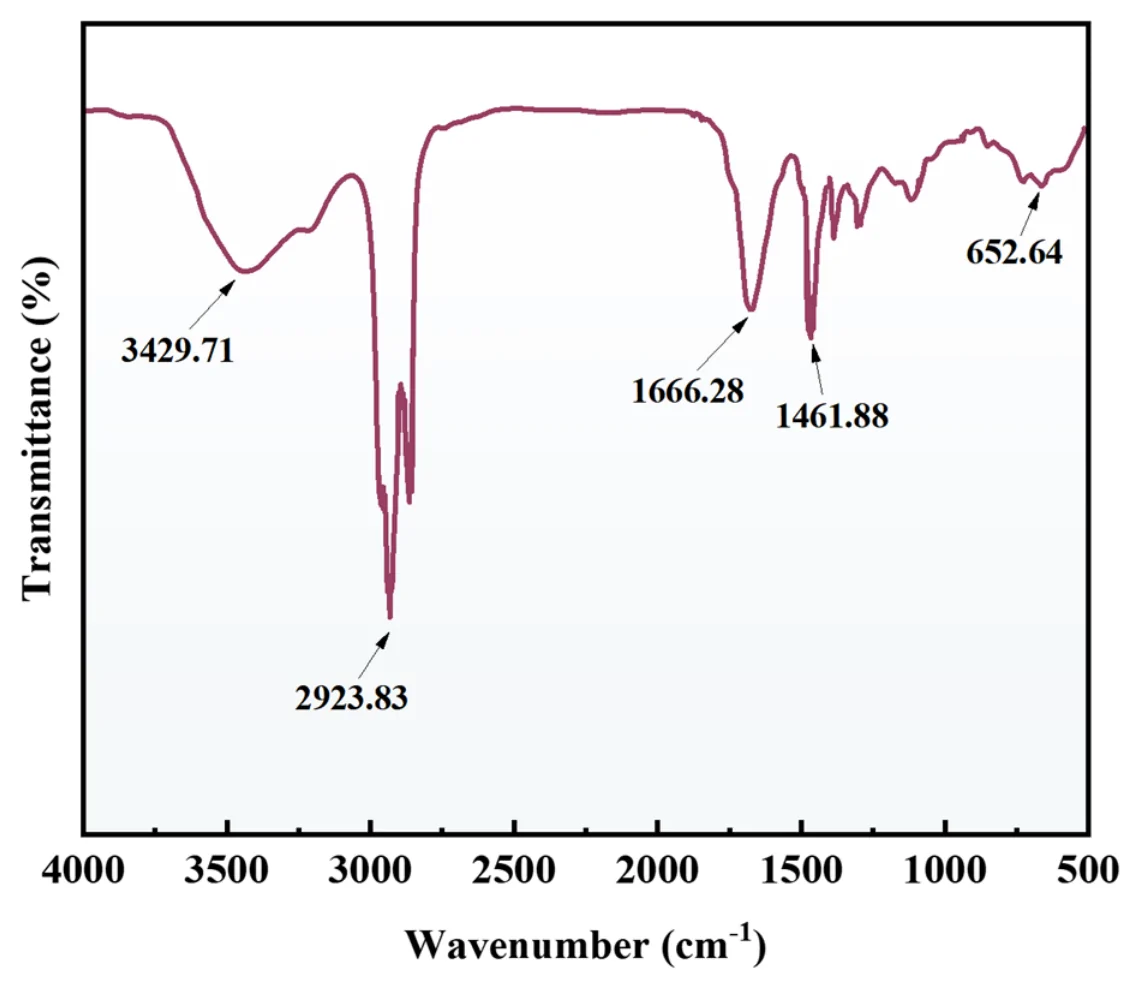
FTIR spectrum of OSGP.

The FTIR spectrum between 500 and 4000 cm^−1^ contained bands assigned to polar groups, alkyl segments, carbonyl structures, and possible ring-related vibrations (Figure 4).

The broad band reported near 3429.71 cm^−1^ was assigned to O-H or N-H stretching, indicating polar groups. The band at 2923.83 cm^−1^ was assigned to the C-H stretching vibration of CH_3_ and CH_2_ groups in the alkyl segments. The band at 1666.28 cm^−1^ was assigned to C=O stretching, and the band at 1461.88 cm^−1^ was assigned to CH_3_/CH_2_ bending. The band at 652.64 cm^−1^ was tentatively assigned to C-H bending or a C-N-related vibration of the pyrrolidone ring. These tentative assignments are summarized in Table 3.

The FTIR spectrum indicates the presence of polar groups, alkyl segments, and carbonyl-containing structures in OSGP. The polar groups may contribute to intermolecular interactions, while the alkyl segments are associated with compatibility with the oil phase. These spectral features are consistent with the functional components used in the OSGP formulation.

### 2.4. Thermal Stability and Oil-Absorption Swelling Behavior

**Figure 5:**
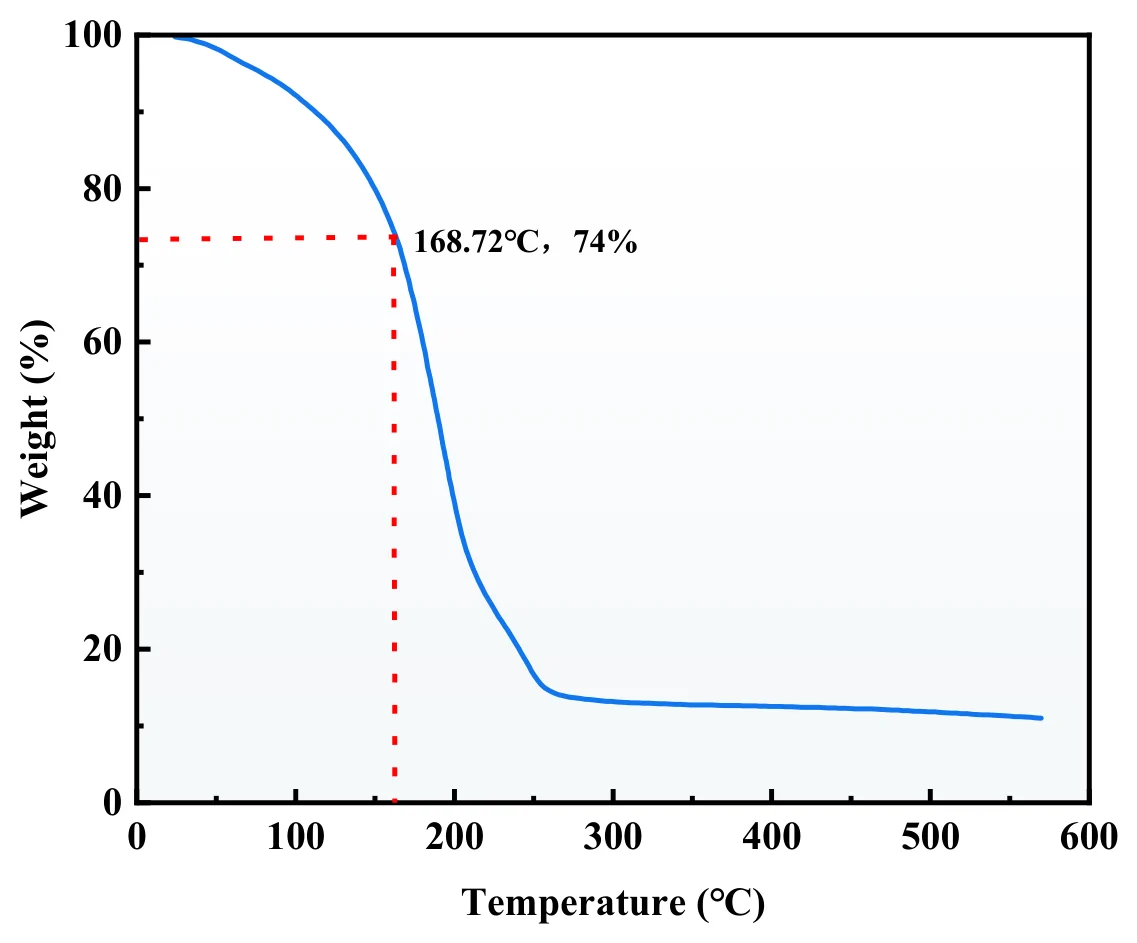
TGA curve of OSGP under nitrogen; the residual mass at 168.72 °C was approximately 74%.

Thermal behavior and oil-absorption swelling influence whether OSGP can retain structure and expand in the tested oil-phase environment. TGA was used to describe mass loss (Figure 5), and swelling tests in white oil at 120 °C were used to determine volume change and swelling kinetics.

The TGA curve showed staged mass loss (Figure 5). At 168.72 °C, OSGP retained approximately 74% of its initial mass (Figure 5). This value was read at a fixed temperature and is reported as residual mass rather than as the decomposition-onset temperature.

Oil-absorption swelling was evaluated using OSGP particles. The initial volume was 31.7 mL and increased to 37.8 mL after 12 h in white oil at 120 °C. The swelling ratio, defined as the swollen volume divided by the initial volume, was 119.2%, corresponding to a volumetric increase of 19.2% (Table 4).

The swelling ratio and percentage volumetric increase were calculated using Equations (1) and (2), respectively.

**Figure 6:**
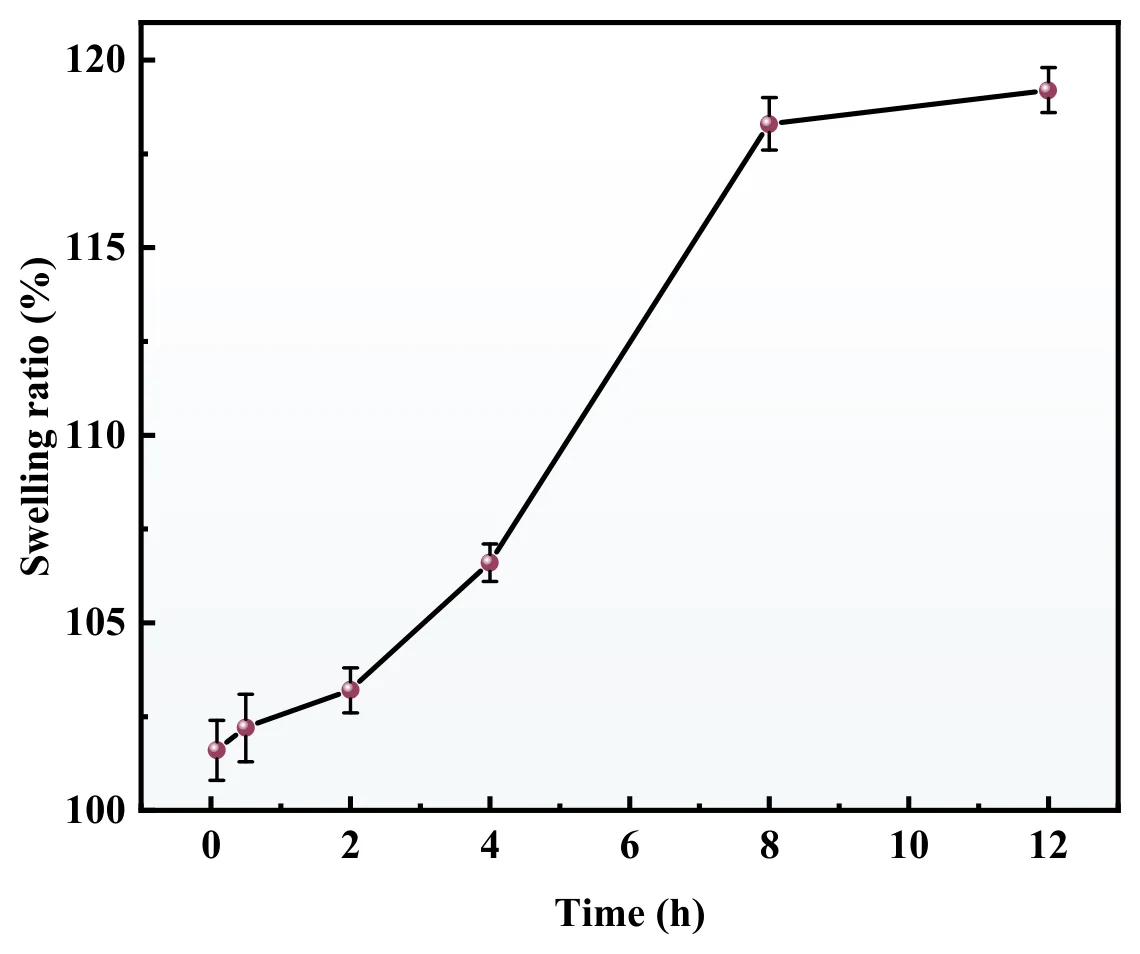
Swelling kinetics of OSGP in white oil at 120 °C. Error bars represent standard deviations (*n* = 3).

Swelling developed in stages (Figure 6). SR increased slowly from 101.6% at 5 min to 103.2% at 2 h, rose to 106.6% at 4 h and 118.3% at 8 h, and reached 119.2% at 12 h. The small difference between 8 and 12 h indicates that swelling approached a plateau after approximately 8 h.

Although the volume increase of OSGP was limited to 19.2%, swelling was not expected to act as the sole sealing mechanism. The 1–3 mm particles first form a bridging and packing structure within the fracture, after which oil-induced swelling can further reduce interparticle voids and improve particle contact. Therefore, moderate swelling may be sufficient when combined with particle bridging and viscoelastic compaction. For comparison, Bai et al. reported a pressure-bearing capacity of 4.7 MPa at 120 °C for an oil-absorbing resin used in 1–3 mm fractures [27], while an oil-absorbing gel reported in another study reached 7.6 MPa in a 3 mm fracture at 140 °C [25]. The present OSGP reached 4.92 MPa in a 3 mm parallel fracture and 8.06 MPa in a wedge-shaped fracture at 120 °C. Although the test conditions and material systems differ, these results indicate that a very large swelling ratio is not necessarily required to obtain measurable fracture-sealing capacity.

The delayed swelling response may reduce premature particle expansion during transport. After initial bridging, continued oil uptake increases the particle volume, narrows interparticle voids, and improves contact with the fracture walls. This behavior forms the basis of the sealing model discussed in Section 2.7.

Overall, OSGP underwent limited and sustained swelling in white oil at 120 °C. OSGP therefore exhibited delayed and sustained swelling, with most of the volumetric increase occurring between 4 and 8 h.

### 2.5. Rheological Properties of OSGP

**Figure 7:**
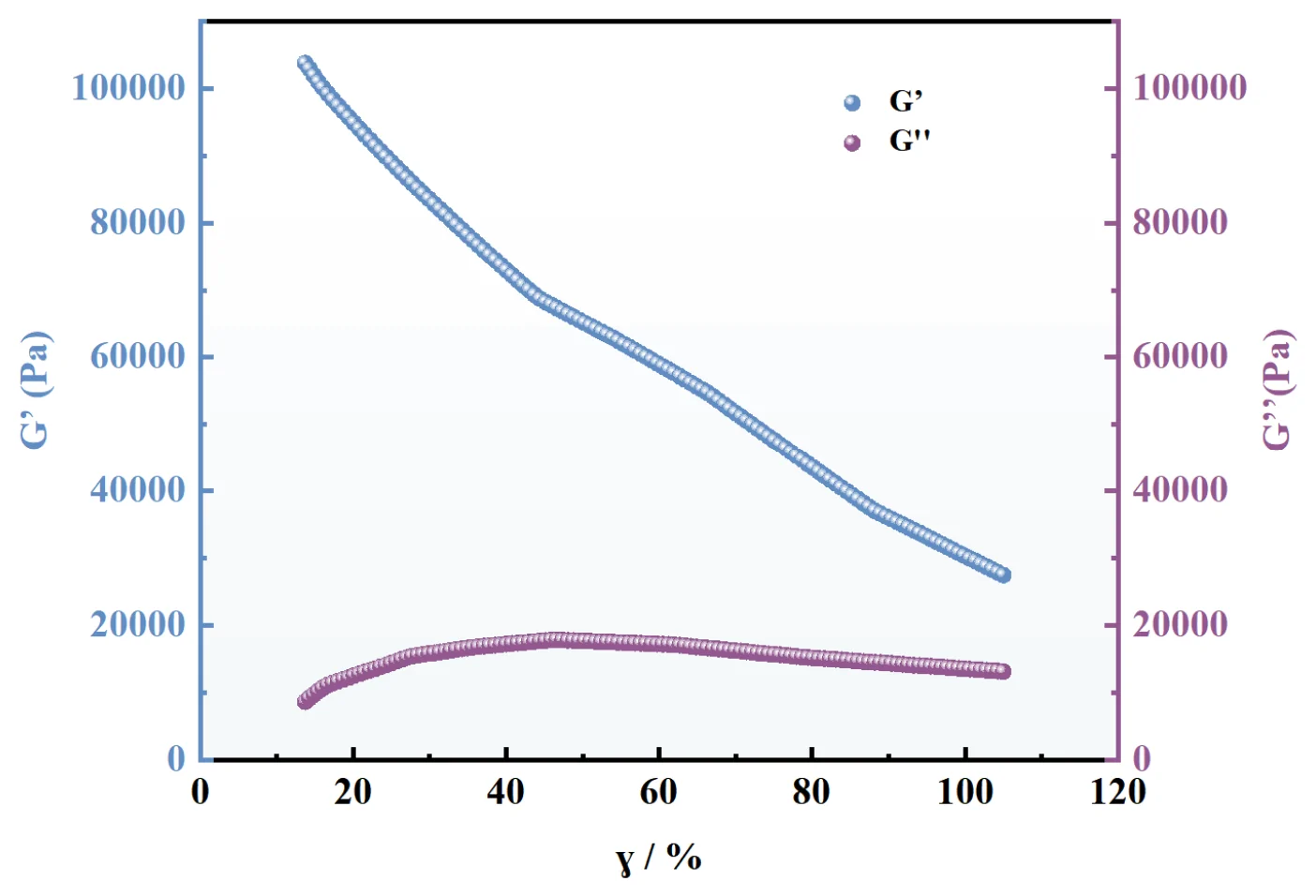
Strain-dependent storage modulus (G′) and loss modulus (G″) of OSGP measured at 25 °C and an angular frequency of 10 rad s^−1^.

Rheological properties were examined to assess the structural stability and deformability of the selected OSGP formulation. A dynamic strain sweep was used to record the storage modulus (G′) and loss modulus (G″) as functions of strain. Figure 7 shows the strain dependence of G′ and G″ for the selected OSGP formulation.

Over the plotted strain range, G′ remained higher than G″, indicating an elastic-dominant response. As strain increased, G′ decreased, whereas G″ initially increased and then gradually decreased. No crossover between G′ and G″ was observed. These results indicate progressive softening of the gel network while the elastic component remained dominant. The results characterize the elastic-dominant and strain-softening behavior of OSGP under the tested room-temperature conditions.

### 2.6. High-Temperature and High-Pressure Fracture-Sealing Performance

High-temperature, high-pressure fracture-sealing tests were used to evaluate OSGP in parallel and wedge-shaped steel fracture models under forward and reverse flow.

#### 2.6.1. Sealing Performance in Parallel Fractures

The parallel-fracture model represented a loss channel with a constant aperture. OSGP particles entered the 3 mm fracture with the oil-phase carrier and accumulated under high-temperature conditions. During subsequent white-oil injection, inlet pressure increased, indicating the formation of a pressure-bearing particle bed.

**Figure 8:**
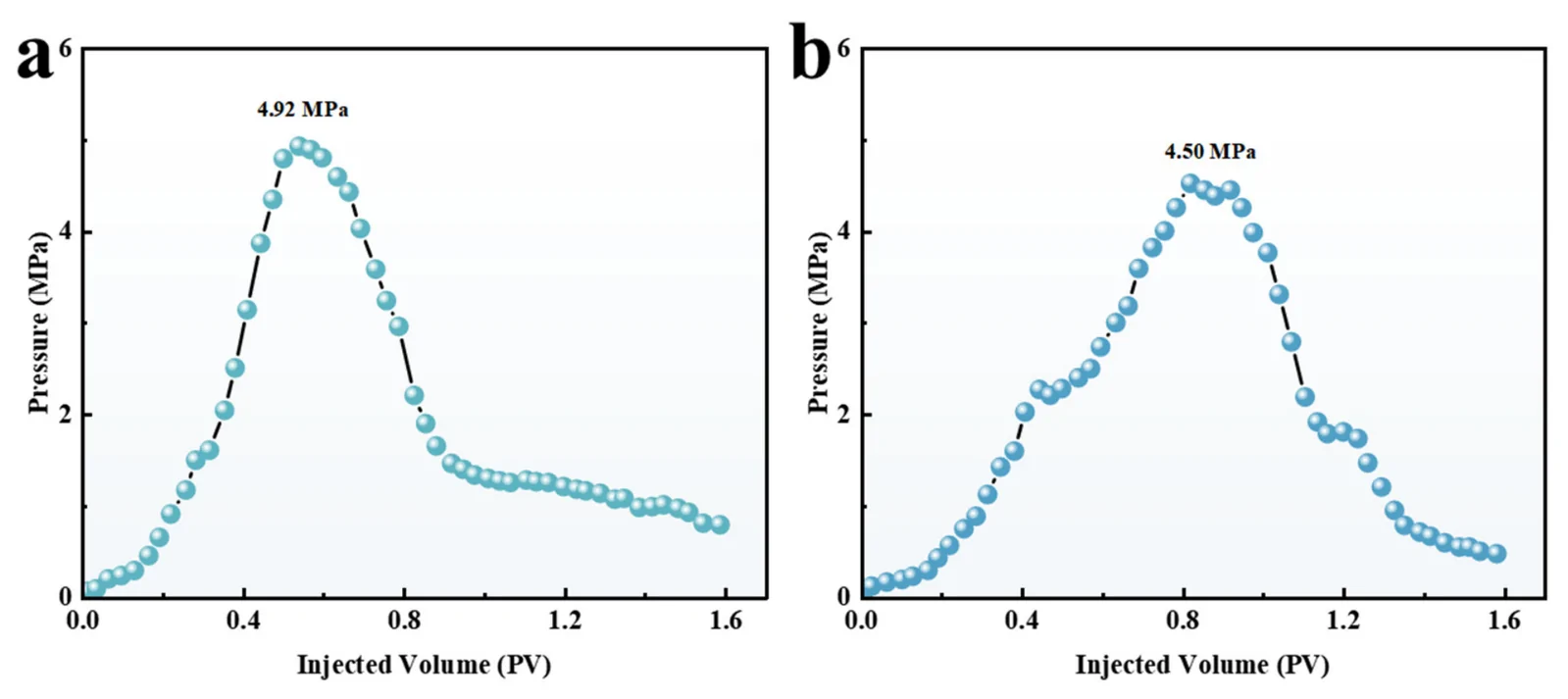
Forward and reverse pressure-bearing curves of OSGP in a 3 mm parallel steel fracture at 120 °C: (**a**) forward loading and (**b**) reverse loading. A 3 wt% OSGP suspension in white oil was used for placement, and white oil was injected at 5.0 mL min^−1^ during the pressure-bearing test.

During forward loading, pressure increased rapidly with injected volume and reached a peak of 4.92 MPa before decreasing sharply and approaching a lower plateau (Figure 8a).

During reverse loading, pressure increased more gradually and reached 4.50 MPa (Figure 8b). The value was slightly lower than the forward peak, but the two values were similar, indicating that the seal retained pressure-bearing capacity when the flow direction was reversed. The similar forward and reverse values indicate limited directional dependence in the parallel fracture.

The parallel-fracture tests therefore demonstrated a pressure-bearing particle bed with similar forward and reverse breakthrough pressures.

#### 2.6.2. Sealing Performance in Wedge-Shaped Fractures

The wedge-shaped model represented a heterogeneous loss channel whose aperture decreased along the flow direction. Relative to a parallel fracture, this geometry promotes particle accumulation and bridging near the narrow region and therefore provides a test of directional fracture adaptation.

**Figure 9:**
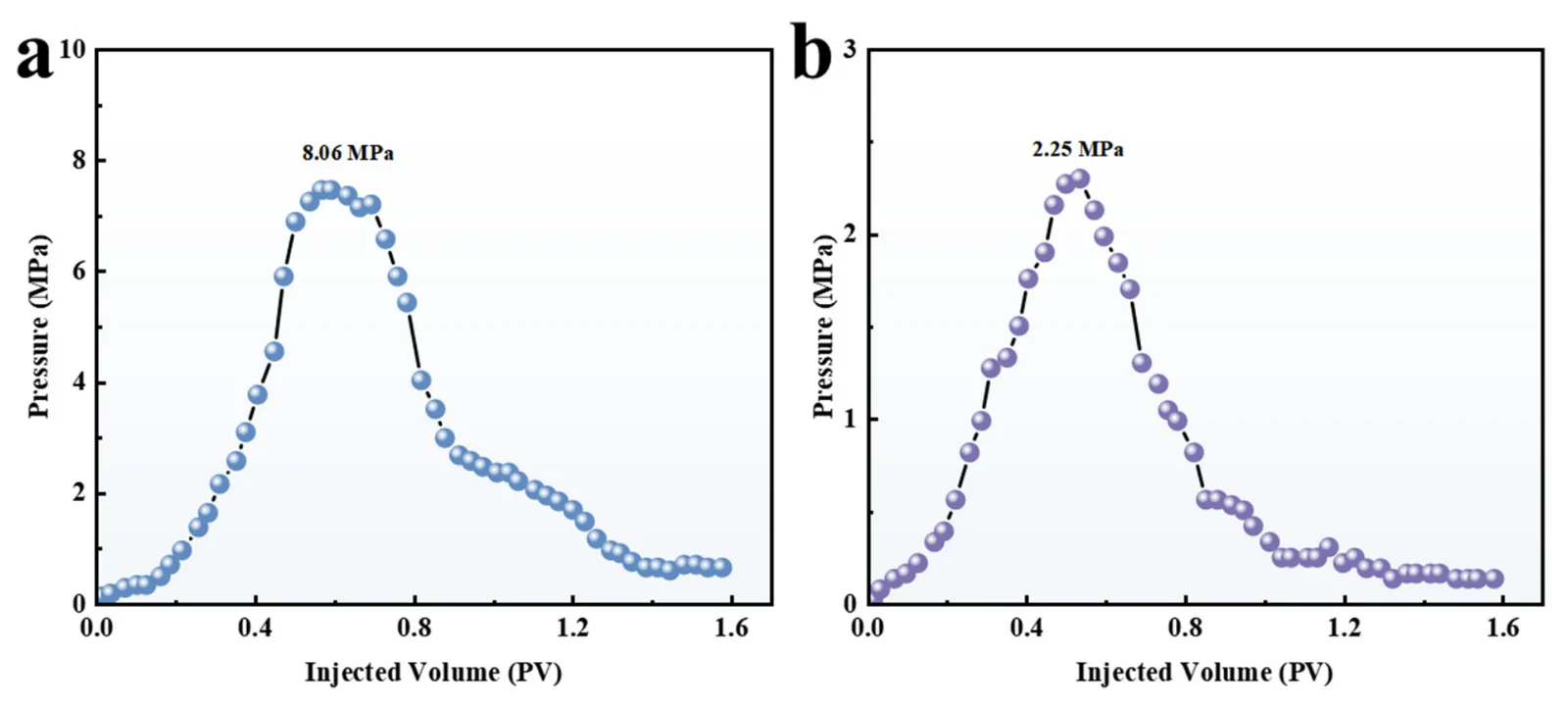
Forward and reverse pressure-bearing curves of OSGP in a wedge-shaped steel fracture with the aperture decreasing from 3 mm to 1 mm at 120 °C: (**a**) forward loading and (**b**) reverse loading. A 3 wt% OSGP suspension in white oil was used for placement, and white oil was injected at 5.0 mL min^−1^ during the pressure-bearing test.

During forward loading, pressure increased rapidly and reached 8.06 MPa (Figure 9a), which was higher than the 4.92 MPa peak in the parallel model. The converging aperture promoted particle accumulation near the constricted region, producing a higher forward breakthrough pressure than that obtained in the parallel fracture (Table 5).

During reverse loading, the peak pressure was 2.25 MPa (Figure 9b), substantially lower than both the forward value for the wedge-shaped fracture and the reverse value for the parallel fracture. The lower reverse pressure indicates that the sealing layer formed in the converging fracture was direction-dependent. When the flow direction was reversed, particle rearrangement, loosening, or partial detachment may have reduced the stability of the packed structure. This directional difference suggests that fracture geometry and flow direction are important factors controlling the pressure-bearing behavior of OSGP.

Fracture geometry and flow direction strongly affected sealing performance. The parallel fracture showed similar forward and reverse peak pressures, whereas the wedge-shaped fracture exhibited a high forward pressure but substantially lower reverse resistance, indicating greater sensitivity to changes in flow direction.

These tests demonstrate the pressure-bearing behavior of OSGP in white oil and artificial steel fractures at 120 °C. Further evaluation in fully formulated oil-based drilling fluids is needed to assess both fracture-sealing performance and the effects of OSGP on drilling-fluid rheology and filtration properties.

Compared with conventional rigid granular lost-circulation materials, which rely mainly on particle-size matching and mechanical bridging within fractures [10,11,12,13,14,15,16,17], OSGP provides an additional oil-responsive deformation mechanism after particle placement. The present results suggest that particle bridging remains the initial step in seal formation, while subsequent oil absorption and particle deformation may further improve contact within the packed layer. Direct quantitative comparison between different studies is difficult because fracture geometry, carrier fluid, particle concentration, and pressure-testing procedures vary considerably.

Nevertheless, the pressure-bearing results obtained in the present study can be placed in the context of previously reported oil-absorbing lost-circulation materials. OSGP reached 4.92 MPa in the 3 mm parallel fracture and 8.06 MPa in the wedge-shaped fracture at 120 °C, which are comparable in magnitude to pressure-bearing values reported for related oil-absorbing gels and resins [25,27]. Because the experimental conditions differ among these studies, these values are used only to provide a performance reference rather than to establish a direct ranking of the materials.

### 2.7. Proposed Sealing Model

**Figure 10:**
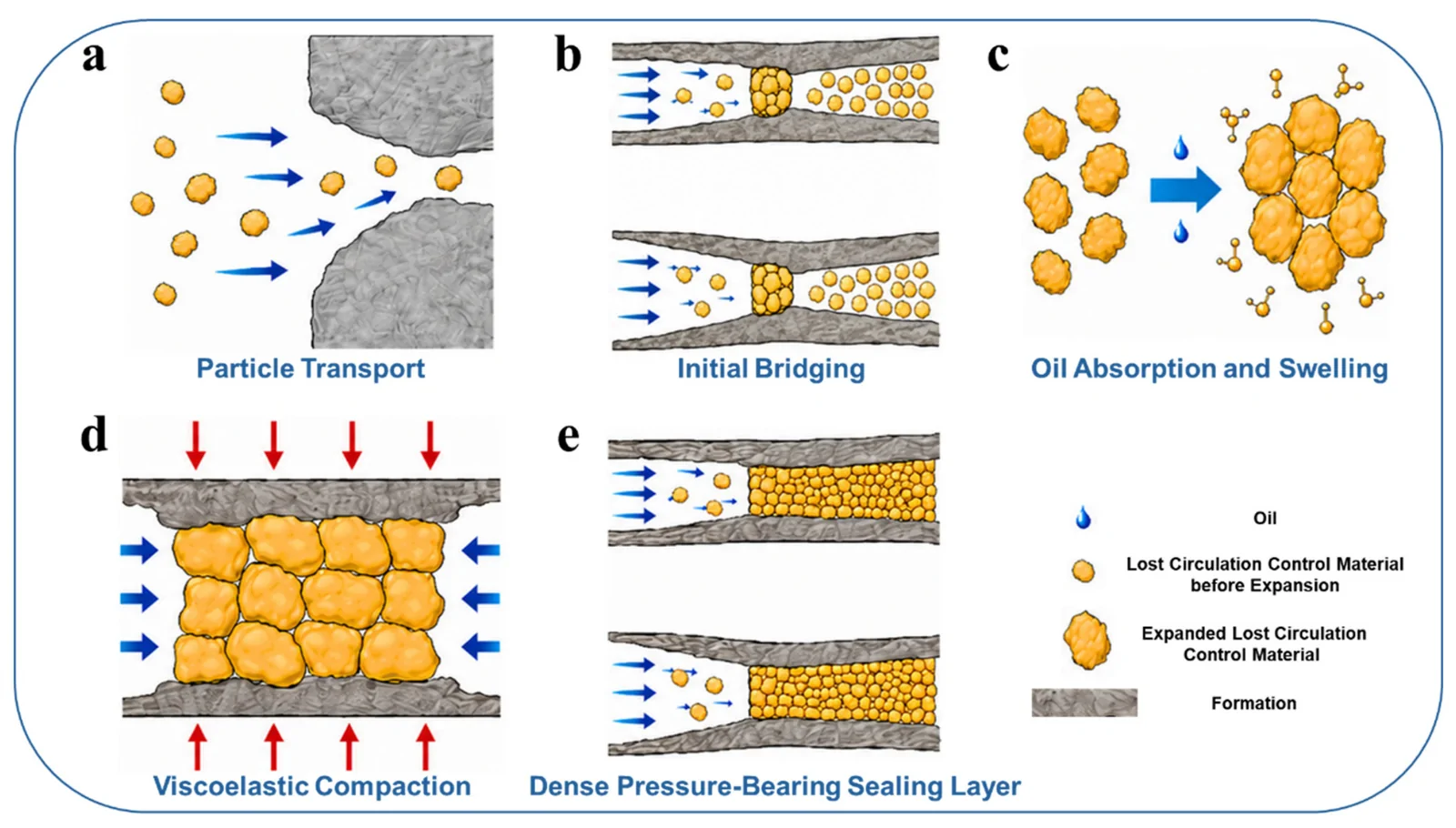
Conceptual sealing model for OSGP in an artificial fracture: (**a**) particle transport in an oil-phase carrier; (**b**) initial bridging and accumulation; (**c**) oil absorption and swelling; (**d**) viscoelastic compaction and void reduction; and (**e**) formation of a pressure-bearing particle layer.

Figure 10 integrates the characterization and pressure-bearing results into a five-stage sealing model comprising particle transport, initial bridging, oil-induced swelling, viscoelastic compaction, and formation of a dense pressure-bearing layer.

During placement, 1–3 mm OSGP particles are transported into the fracture and accumulate where the aperture narrows or approaches the particle size. Particle contacts form an initial bridging skeleton, particularly near the constricted region of the wedge-shaped fracture. Subsequent oil uptake increases the particle volume and reduces interparticle voids. Under differential pressure, the deformable nature of the gel particles may facilitate particle rearrangement and compaction, while their structural integrity contributes to maintenance of the packed layer.

The similar forward and reverse peak pressures in the parallel fracture indicate relatively low directional dependence, whereas the wedge-shaped fracture shows substantially stronger forward than reverse resistance. These results indicate that the sealing performance depends on both particle deformation and fracture geometry. Overall, initial bridging forms the structural skeleton, oil-induced swelling reduces void space, and viscoelastic deformation promotes formation of a compact pressure-bearing layer.

## 3. Conclusions

An oil-absorbing swelling gel plugging agent (OSGP) was prepared by W/O emulsion free-radical polymerization. Under the tested single-factor conditions, a polymerization temperature of 65 °C and a stirring speed of 800 rpm were selected. After 12 h in white oil at 120 °C, the swollen bulk particle volume reached 119.2% of the initial volume, corresponding to a net volume increase of 19.2%.

SEM showed that oil exposure followed by drying produced a smoother external surface without obvious particle collapse or fragmentation. FTIR indicated the presence of polar, alkyl, and carbonyl-containing structures, while TGA characterized the staged thermal mass-loss behavior of OSGP. At 25 °C, G′ remained higher than G″ over the plotted strain range, indicating an elastic-dominant response with progressive strain-induced softening.

In independent forward and reverse pressure-bearing tests at 120 °C, the breakthrough pressures were 4.92/4.50 MPa in the 3 mm parallel fracture and 8.06/2.25 MPa in the 3 mm-to-1 mm wedge-shaped fracture, respectively. The marked directional difference in the wedge-shaped fracture demonstrates the important effects of fracture geometry and flow direction on sealing stability. The combined observations are consistent with a proposed sealing process involving initial particle bridging, oil-induced swelling, particle deformation, and compaction. Further work should evaluate high-temperature rheological behavior, compatibility with fully formulated oil-based drilling fluids, sealing in rock fractures, and durability under cyclic or repeated-flow conditions, together with post-test characterization of the sealing layer.

## 4. Materials and Methods

### 4.1. Materials

N-vinylpyrrolidone (NVP, ≥98%) and divinylbenzene (DVB, ≥96%) were purchased from Shanghai Aladdin Biochemical Technology Co., Ltd. (Shanghai, China). The functional acrylate monomer QW-1 (≥98%) was purchased from Shanghai Aladdin Biochemical Technology Co., Ltd. (Shanghai, China). SMA (≥96%) was purchased from Boxin Chemical Raw Materials (Shanghai, China). AIBN (≥97%), liquid paraffin, Span 80, and bentonite were purchased from Sinopharm Chemical Reagent Co., Ltd. (Shanghai, China). Tween 80 was purchased from Shanghai Macklin Biochemical Co., Ltd. (Shanghai, China), PEG600 was purchased from Shanghai Aladdin Biochemical Technology Co., Ltd., and white oil (No. 5 industrial grade; kinematic viscosity: 4.14–5.06 mm^2^·s^−1^ at 40 °C; density: approximately 0.82 g·cm^−3^ at 20 °C) was purchased from Shandong Xinbaiwei Chemical Co., Ltd. (Shandong, China). Deionized water was produced in the laboratory. All reagents were used without further purification; the materials are summarized in Table 6.

### 4.2. Preparation of OSGP

OSGP was prepared by W/O emulsion free-radical polymerization. White oil (80 g) and liquid paraffin (3 g) were first added to a three-neck flask and mechanically stirred at 50 °C until a homogeneous oil phase formed. Span 80 and Tween 80 were then added as emulsifying/dispersing agents and stirred until dissolved. SMA was subsequently added and dispersed uniformly in the oil phase.

After the oil phase cooled to room temperature, deionized water (20 g) was added slowly under stirring to form a W/O emulsion. NVP and QW-1 were then added and dispersed. PEG600 and bentonite were introduced, and the mixture was sonicated at 40 °C and 200 W for 5 min to improve dispersion of the pore-forming and inorganic reinforcing components.

DVB and AIBN were added, and the mixture was stirred for 15 min. Nitrogen was introduced to remove oxygen and reduce oxygen inhibition of free-radical polymerization. The reaction was maintained at 65 °C for 8 h to promote polymerization, crosslinking, and formation of a bulk gel.

The gel was cut into pieces and dried under vacuum at 60 °C for 24 h. The dried gel was coarsely crushed and classified using standard sieves. Particles passing through a 3 mm aperture sieve and retained on a 1 mm aperture sieve were collected as the 1–3 mm fraction for subsequent tests.

The selected formulation comprised 20 g NVP, 10 g QW-1, 12 g SMA, 5 g DVB, 0.4 g AIBN, 6 g PEG600, 0.6 g bentonite, 3 g Span 80, 0.3 g Tween 80, and 3 g liquid paraffin. The selected curing temperature and stirring speed were 65 °C and 800 rpm, respectively.

### 4.3. Single-Factor Screening of Formulation and Preparation Parameters

Single-factor experiments were conducted to examine the effects of monomer composition, DVB dosage, Tween 80 dosage, bentonite dosage, polymerization temperature, and stirring speed on the swelling behavior of OSGP. In each experimental series, only one factor was varied while the remaining formulation components and preparation conditions were kept unchanged. The swelling ratio in white oil at 120 °C was used as the primary screening index because oil-induced volume expansion is a key functional response of OSGP after entering a fracture. The tested levels of each factor and the corresponding selected conditions are summarized in Table 2. The selected formulation was subsequently subjected to rheological characterization and fracture-sealing tests.

### 4.4. Structural Characterization

Dry OSGP particles and post-swelling dried particles were mounted on conductive adhesive, coated with an approximately 10 nm gold layer, and observed using a Quanta FEG 250 scanning electron microscope (FEI, Hillsboro, OR, USA) at an accelerating voltage of 6.00 kV and a magnification of 5000×. Five randomly selected fields of view were examined for each sample. After swelling, the particles were cooled to room temperature, filtered, gently blotted to remove surface oil, and vacuum-dried at 40 °C before SEM observation.

FTIR spectra were collected using the KBr-pellet method. Dried OSGP and dry KBr were mixed at an approximate mass ratio of 1:100, ground thoroughly, pressed into a transparent pellet, and scanned from 500 to 4000 cm^−1^. The spectra were recorded using a Nicolet iS10 FTIR spectrometer (Thermo Fisher Scientific, Waltham, MA, USA) at a spectral resolution of 4 cm^−1^, with 32 accumulated scans per spectrum.

TGA was performed under nitrogen by heating dried OSGP from 20 to 600 °C at 20 °C min^−1^. The residual mass at 168.72 °C was read directly from the curve; no separate onset-temperature criterion was applied. Measurements were performed using a TGA 550 thermogravimetric analyzer (TA Instruments, New Castle, DE, USA), with approximately 8–10 mg of sample and a nitrogen flow rate of 50 mL min^−1^. Three independent measurements were conducted, and a representative curve is presented.

### 4.5. Oil-Absorption Swelling Test

White oil was used as the swelling medium. The dry OSGP used in the test was the 1–3 mm fraction obtained by sieving. A known bulk volume of the dry particles was recorded as V_0_, after which the particles were immersed in excess white oil and aged at 120 °C for 12 h. The sample was removed and placed on a screen lined with oil-absorbing paper to remove excess surface oil, and the bulk volume of the swollen particles, V_t_, was measured by liquid displacement.

The swelling ratio (SR) was defined as the swollen particle volume relative to the initial dry particle volume and was calculated using Equation (1):

(1) SR = V_t_/V_0_ × 100%

where V_0_ is the initial volume of the dry OSGP particles and V_t_ is the particle volume after oil absorption. An SR of 100% therefore represents no volume change, whereas an SR greater than 100% indicates swelling.

For kinetic measurements, OSGP was aged in white oil at 120 °C and sampled after 5 min, 30 min, 2 h, 4 h, 8 h, and 12 h. Excess surface oil was removed at each time point and the particle volume was measured. Independent portions from the same batch were used at each time point.

The net percentage increase in particle volume was calculated separately using Equation (2):

(2) Volume increase = (V_t_ – V_0_)/V_0_ × 100%

### 4.6. Rheological Test

Dynamic rheology was used to characterize the strain-dependent viscoelastic response of the selected OSGP formulation. Rheological specimens were prepared from the bulk gel obtained after polymerization and crosslinking, before vacuum drying, crushing, and sieving. The bulk gel was cut into disk-shaped specimens approximately 25 mm in diameter and 1.0 mm in thickness. Measurements were performed using an MCR 302 rotational rheometer (Anton Paar GmbH, Graz, Austria) equipped with a 25 mm parallel-plate fixture. Before testing, each sample was equilibrated at 25 °C for 5 min. A strain sweep was then conducted at an angular frequency of 10 rad s^−1^ over a programmed strain range of 0.01–100%, and G′ and G″ were recorded as functions of strain. The temperature-control accuracy was ±0.1 °C, and three replicate measurements were conducted for each sample.

### 4.7. High-Temperature and High-Pressure Fracture-Sealing Test

A high-temperature, high-pressure displacement apparatus was used to evaluate pressure-bearing fracture sealing. Two steel fracture models were tested: a 3 mm × 3 mm × 20 cm parallel fracture and a wedge-shaped fracture with the aperture decreasing from 3 mm to 1 mm along a length of 20 cm. The temperature was set to 120 °C. The effective fracture volumes were approximately 1.8 mL for the parallel fracture and 1.2 mL for the wedge-shaped fracture. The 1–3 mm OSGP particle fraction was selected so that the particle size was comparable to the characteristic aperture of the fracture models. In the parallel fracture, this particle-size range was expected to favor initial bridging and subsequent packing, whereas in the wedge-shaped fracture, particles could be progressively retained as the aperture decreased from 3 mm to 1 mm.

Approximately 100 mL of the 3 wt% OSGP suspension, corresponding to 2.46 g of OSGP, was used for each test. Before the pressure-bearing stage, the inlet pressure was approximately 0 MPa. The steel fracture models were reused after each test following disassembly, thorough cleaning, drying, and reassembly.

Before each test, the fracture model was installed in a core holder, the lines were connected, and the confining pressure was increased to 10 MPa. White oil was injected at 9.0 mL min^−1^ until continuous effluent was observed. A 3 wt% OSGP particle suspension in white oil was then placed in a stirred intermediate vessel and agitated at 200–300 rpm to maintain dispersion.

The white-oil line was closed, and the OSGP suspension was injected at 9.0 mL min^−1^ until continuous suspension exited the model. Excess suspension in the inlet line was then discharged, the lines were closed, and the model was then held at 120 °C for 12 h to allow sufficient oil absorption and swelling before the pressure-bearing test.

The forward and reverse pressure-bearing tests were conducted independently. For each test, the fracture model was cleaned and reassembled, and a new OSGP sealing layer was formed following the same placement and 12 h aging procedure described above. For the forward pressure-bearing test, white oil was injected in the same direction as OSGP placement at 5.0 mL min−1, and the inlet pressure was recorded continuously. For the reverse pressure-bearing test, a separately prepared sealing layer was used, and white oil was injected from the direction opposite to OSGP placement at the same flow rate. When the pressure reached a peak and then decreased rapidly, the seal was considered to have been breached, and the maximum pressure immediately before the abrupt decrease was recorded as the breakthrough pressure.

After testing, heating was stopped, confining pressure was released, and the fracture model was removed. The maximum pressure recorded immediately before the abrupt pressure decrease was defined as the breakthrough pressure.

## Figures and Tables

**Table 1:** Selected formulation and preparation conditions of OSGP.

Component or Condition	Function	Selected Value
NVP	Functional monomer providing polar groups and polymerization reactivity	20 g
QW-1	Functional acrylate monomer	10 g
SMA	Oil-affinitive network component	12 g
DVB	Crosslinker for the three-dimensional network	5 g
AIBN	Free-radical initiator	0.4 g
PEG600	Pore-forming/structure-regulating component	6 g
Bentonite	Inorganic reinforcing component	0.6 g
Span 80	Oleophilic dispersant	3 g
Tween 80	Co-dispersant	0.3 g
Liquid paraffin	Oil-phase regulating component	3 g
Stirring speed	Controls emulsion dispersion and gel structure	800 rpm
Curing temperature	Controls polymerization and gelation	65 °C

**Table 2:** Summary of the single-factor screening ranges and selected conditions for OSGP.

Factor	Tested Range	Main Effect	Selected Condition
Monomer composition	Different NVP/QW-1/SMA ratios	Oil affinity, network flexibility, and swelling	20 g/10 g/12 g
DVB dosage	1–7 g	Crosslink density and network stability	5 g
Tween 80 dosage	0–0.3 g	W/O emulsion stability and network uniformity	0.3 g
Bentonite dosage	0.2–1.5 g	Inorganic reinforcement and structural support	0.6 g
Curing temperature	50–95 °C	Polymerization rate and network uniformity	65 °C
Stirring speed	200–1100 rpm	Emulsion dispersion and network formation	800 rpm

**Table 3:** Principal FTIR absorption bands and their assignments for OSGP.

Wavenumber (cm^−1^)	Possible Assignment	Structural Interpretation
3429.71	O–H or N–H stretching	Polar groups and possible intermolecular interactions
2923.83	CH3/CH2 stretching	Alkyl segments associated with oil affinity
1666.28	C=O stretching	Carbonyl-containing structures in the network
1461.88	CH3/CH2 bending	Additional evidence of alkyl structures
652.64	C–H bending or C–N-related vibration	Possible ring-related or NVP-derived structural unit

**Table 4:** Swelling behavior of OSGP in white oil at 120 °C.

Initial Volume, V_0_ (mL)	Swollen Volume, V_t_ (mL)	Swelling Ratio, SR (%)	Volume Increase (%)
31.7	37.8	119.2	19.2

**Table 5:** High-temperature and high-pressure fracture-sealing performance of OSGP.

Fracture Model	Pressure Direction	Peak Pressure (MPa)	Pressure Response
3 mm parallel	Forward	4.92	Rapid increase followed by pressure decrease
3 mm parallel	Reverse	4.50	Gradual increase followed by pressure decrease
3 × 1 mm wedge-shaped	Forward	8.06	Rapid pressure buildup
3 × 1 mm wedge-shaped	Reverse	2.25	Gradual pressure buildup

**Table 6:** Materials used for the preparation and evaluation of OSGP.

Material	Abbreviation	Purity	Function
N-vinylpyrrolidone	NVP	≥98%	Functional monomer
Acrylate monomer QW-1	QW-1	≥98%	Functional monomer
Styrene–maleic anhydride copolymer	SMA	≥96%	Oil-affinitive network component
Divinylbenzene	DVB	≥96%	Crosslinker
Azobisisobutyronitrile	AIBN	≥97%	Initiator
Polyethylene glycol 600	PEG600	≥96%	Pore-forming/structure regulator
Bentonite	—	≥99%	Inorganic reinforcement
Span 80	—	≥98%	Dispersant
Tween 80	—	≥98%	Dispersant
Liquid paraffin	—	≥99%	Oil-phase regulator
White oil	—	≥99%	Oil-absorption medium

## Data Availability

Data is contained within the article.
